# Impact of Sex on the Association between Flexibility and Arterial Stiffness in Older Adults

**DOI:** 10.3390/medicina58060789

**Published:** 2022-06-12

**Authors:** Tae-Kyung Yoo, Soo-Hyun Park, Sae-Jong Park, Jong-Young Lee

**Affiliations:** 1 Department of Medicine, MetroWest Medical Center, Framingham, MA 01702, USA; tae_kyung.yoo@tufts.edu; 2 Department of Sport Science, Korea Institute of Sport Science, Seoul 01794, Korea; otajulia@kspo.or.kr; 3 Division of Cardiology, Department of Internal Medicine, Kangbuk Samsung Hospital, Sungkyunkwan University School of Medicine, Seoul 03181, Korea

**Keywords:** baPWV, flexibility, arterial stiffness, older adults

## Abstract

*Background and Objectives*: Flexibility is one of the most important physical fitness parameters in the geriatric population. Brachial–ankle pulse wave velocity (baPWV) is a measure of systemic arterial stiffness. However, data on the association between flexibility and arterial stiffness in the older adult population are limited. Therefore, we aim to investigate this association by using the sit-and-reach test (SRT) and measuring baPWV. *Materials and Methods*: We reviewed data from the 2014–2015 Korean Institute of Sports Science Fitness Standards Project. Individuals older than 65 years with SRT and baPWV data were included. A generalized linear regression analysis was conducted to assess the association between flexibility and arterial stiffness. Multiple relevant confounding factors were adjusted. *Results*: A total of 615 individuals were included in the analysis. The mean age of the male and female participants was 71.44 ± 4.42 and 70.64 ± 4.13 years, respectively. The mean SRT result was 6.58 ± 9.97 and 18.04 ± 7.48 cm, respectively. After multivariable adjustment among the male participants, the higher SRT result tertiles were inversely associated with baPWV (β (95% confidence interval): 3.11–11.00 cm, −74.45 (−140.93, −8.55); ≥11.01 cm, −108.17 (−177.65, −38.70)) in comparison with the lowest tertile. The female participants did not show any significant correlation between the SRT result and baPWV. *Conclusions*: Our results suggest an inverse association between trunk flexibility and systemic arterial stiffness, expressed as the SRT result and baPWV, respectively, in older Korean men but no association in older Korean women. Sex differences might influence the association between flexibility and arterial stiffness in the older adult population.

## 1. Introduction

Flexibility is one of the most important physical fitness parameters in the geriatric population as it is associated with functional autonomy and the performance of the activities of daily living [1]. In addition, it has been considered a major factor in the preventive treatment of musculotendinous strains [2]. However, age-related alterations in the skeletal muscles and connective tissues diminish flexibility [2,3]. This results in difficulties in performing the activities of daily living and functioning in the geriatric population [3].

Recently, the impact of aortic stiffness in the development of cardiovascular disease has been highlighted. It has been identified as an independent risk factor for mortality and cardiovascular disorders [4]. Brachial–ankle pulse wave velocity (baPWV) is frequently used to measure arterial stiffness as it is relatively simple and convenient [5]. In addition, it provides information similar to central pulse wave velocity and reflects systemic arterial stiffness [6,7].

Interestingly, several studies have suggested a relationship between flexibility and systemic arterial stiffness [2,8,9]. Arterial stiffness is determined by the intrinsic elastic properties of the smooth muscles and connective tissues in the artery [2]. Similar to flexibility, arterial stiffness aggravates over time owing to the fracture and fragmentation of the elastin fibers and age-related changes from a cellular ionic basis [10]. In addition to aging, another aspect to consider in arterial stiffness is sex differences. Before the premenopausal period, arterial stiffness in women is significantly lower than in age-matched men; however, the difference disappears upon reaching menopause [9]. This change in arterial stiffness in older adults poses a difficulty in applying the result of a previous study in which only small numbers of older adults were included [2]. In addition, another study suggested potential sex differences in the relationship between flexibility and arterial stiffness. However, the exact impact of sex differences in this relationship is not completely elucidated. Moreover, study data on such relationships in older adults are even more limited.

Therefore, we aim to investigate the sex differences in relation to the association between flexibility and arterial stiffness, assessed on the basis of the SRT result and baPWV, respectively, in an older Korean adult population.

## 2. Materials and Methods

### 2.1. Study Design and Cohort

We reviewed data from the 2014–2015 Korean Institute of Sports Science Fitness Standard (KISS FitS) Project [11]. The KISS FitS Project was started in 2011 by the Korea Sports Promotion Foundation and the Ministry of Culture and Tourism. It was a nationwide project to evaluate physical fitness levels and facilitate a healthy lifestyle among the general Korean population [12]. The study participants were recruited from the general population, and physical fitness tests were conducted at the strength certification centers in Korea [13].

This study evaluates raw data from the KISS FitS Project [14]. Individuals older than 65 years with SRT and baPWV data were included. This study was approved by the research ethics committee of the Korean Institute of Sports Science (No. KISS-201504-EFS-001-01, approval date 4 May 2015). Participants provided informed consent during the recruitment process.

### 2.2. Covariates

During the physical fitness test, baseline data were collected. Baseline demographic data and medical history, including age, history of smoking, physical activity level, history of hypertension, history of dyslipidemia, history of stroke, and history of myocardial infarction, were collected through interviews. The participants were considered smokers if they smoked cigarettes at the time of the interview and had smoked more than 100 cigarettes in their lifetime [15]. The participants were defined as physically active if they engaged in more than 150 min of moderate-intensity exercise (4.0 METs) or the equivalent combination in a week [16]. Anthropometric data, including weight (kg) and height (cm), were measured in a standardized manner. A stadiometer (Seca, Seca Corporation, Columbia, MD, USA) and an electronic weight scale (Inbody 720, Biospace, Seoul, Korea) were used to measure their height and weight. Thereafter, the body mass index (BMI; kg/m^2^) was calculated. Blood pressure and resting heart rate were measured after resting for at least 15 min in a sitting position using an automated sphygmomanometer (HEM-9000AI, Omron, Japan) [17]. All participants were asked to fast for at least 12 h before blood sample collection. Blood specimens were collected from the antecubital vein using a heparinized syringe.

### 2.3. SRT

To perform the SRT, the participants sat on the floor with both knees straight and their feet bare. The soles of their feet were placed on the measuring instrument. The participants were asked to stretch out their hands to the front. When the examiner provided the start signal, the participants lowered their upper bodies and used their fingertips to push the measurement plate as far as possible. The distance from the starting point and the location of the measurement plate was measured twice, and the maximum value was recorded in units of 0.1 cm [13].

### 2.4. BaPWV Measurement

The participants were asked to continue home medications and fast for at least 4 h before the measurement. They rested for at least 5 min before the baPWV measurement. They were placed in a supine position, with cuffs placed on bilateral arms and ankles. An electrocardiogram (EKG) lead was attached to bilateral wrists to monitor the EKG findings, and a microphone was placed on the left sternal side of the second intercostal space. The baPWV was then recorded using a VP-1000 instrument (Colin Co., Komak, Japan), which measures bilateral brachial and posterior tibial artery pressure waveforms using an oscillometric method. It was calculated automatically for each arterial segment as the path length divided by the corresponding time interval. The sampling time was 10 s for each pulse wave velocity measurement, and baPWV was measured twice on each side. The average value was recorded for each side [5,18]. The coefficients of variation for the left and right baPWVs were 12.3% and 12.6%, respectively. We used the mean baPWV of the left and right baPWVs for the analyses.

### 2.5. Statistical Analysis

The degree of linear association was assessed using the Pearson correlation test. The linear relationship between baPWV and the SRT result was illustrated using scatterplots. The participants were categorized into tertiles according to the SRT result, owing to the skewed distribution. The chi-square test was used to compare categorical variables, and one-way analysis of variance was used to compare continuous variables in each tertile. Means plots were used to graphically illustrate the relationship between the mean baPWV and the SRT result.

The association between the SRT result and baPWV was assessed using a generalized linear model with adjustments for confounding factors. The adjusted model was adjusted for age, history of smoking, physical activity level, history of hypertension, history of dyslipidemia, history of stroke, history of myocardial infarction, BMI, serum glucose level, serum low-density lipoprotein (LDL) cholesterol level, systolic blood pressure, and heart rate at rest. The lowest tertile of the SRT result was used as the reference. Beta coefficients were used to assess the association between the SRT result and baPWV, and 95% confidence intervals (CIs) were calculated. Two-tailed *p*-values of <0.05 were considered statistically significant. All statistical analyses and graph formations were performed using IBM SPSS version 28.0 (IBM Statistics, IBM Co., Chicago, IL, USA).

## 3. Results

### 3.1. Baseline Characteristics

Initially, 683 participants were included. Then, participants with missing data for the analysis were excluded (*n* = 68; BMI, *n* = 5; LDL cholesterol level, *n* = 63, glucose level, *n* = 63, history of smoking, *n* = 10; physical activity level, *n* = 10; and history of myocardial infarction, *n* = 6). Ultimately, a total of 616 (men, *n* = 308; women, *n* = 308) participants were included in the analysis.

The mean age of the male and female participants was 71.44 ± 4.42 and 70.64 ± 4.13 years, respectively. The mean SRT result was 6.58 ± 9.97 and 18.04 ± 7.48 cm, respectively. The tertiles ranged as follows: ≤3.10, 3.11–11.00, and ≥11.01 cm for men (total range, −24–29.20 cm); ≤15.00, 15.01–22.00, and ≥22.01 cm for women (total range, −6.8–32.10 cm).

Participants’ baseline characteristics are expressed according to SRT tertile. In the male participants, there was a significant difference in age (*p* = 0.003) and physical activity level (*p* = 0.009) between each SRT result tertile. Meanwhile, there was no significant difference in height, weight, BMI, LDL cholesterol level, serum glucose level, systolic and diastolic blood pressures, smoking rate, and prevalence of the history of hypertension, dyslipidemia, smoking, stroke, and myocardial infarction between each tertile.

In the female participants, there was a significant difference in age (*p* = 0.003) and prevalence of the history of hypertension (*p* = 0.038) between each SRT result tertile. Conversely, there were no significant differences in all other characteristics (Table 1).

### 3.2. Association between BaPWV and the SRT Result

**Figure 1:**
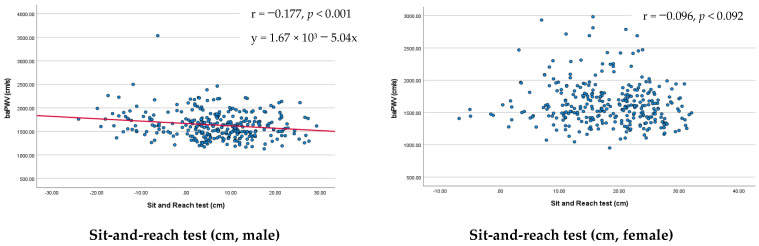
Association between baPWV and the sit-and-reach test: male/female participants’ results, expressed as scatter plots. Blue dots: individual data point. Red line: linear relationship between baPWV and sit-and-reach test result. baPWV, brachial–ankle pulse wave velocity.

Before dividing it into tertiles, the linear association between baPWV and the SRT result was assessed. In male participants, baPWV and the SRT result showed a significant linear association (r = −0.177, y = 1.67 × 10^3^ − 5.04x, *p* < 0.001). However, in female participants, baPWV and the SRT result did not show a significant association (r = −0.096, *p* < 0.092; Figure 1).

In the male participants, the mean baPWV was 1635.64 ± 283.52 cm/s (range, 1130.00–3535.50 cm/s). The mean baPWV for the lowest, middle and highest SRT result tertiles was 1713.49 ± 331.31, 1608.04 ± 260.95, and 1595.36 ± 246.63 cm/s, respectively (*p* = 0.006). The mean baPWV showed a decreasing trend with better performance in the SRT (*p*_trend_ = 0.003).

**Figure 2:**
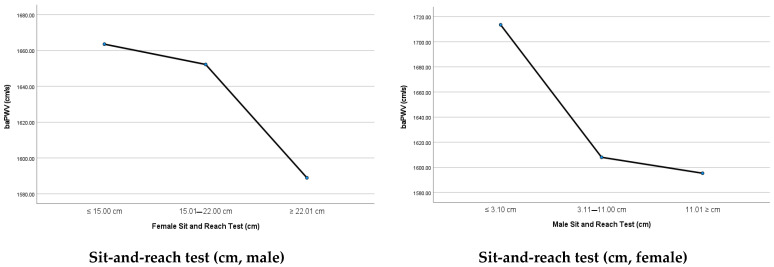
Comparison of the mean baPWV in each sit-and-reach test result tertile using mean plots. baPWV, brachial–ankle pulse wave velocity.

In the female participants, the mean baPWV was 1635.09 ± 333.89 cm/s (range, 951.0–2983.50 cm/s). The mean baPWV for the lowest, middle and highest SRT result tertiles was 1663.54 ± 344.91, 1652.23 ± 358.52, and 1588.95 ± 292.79 cm/s, respectively (*p* = 0.227). The trend of the baPWV, according to the SRT result tertiles, was not significant (*p*_trend_ = 0.109; Table 2 and Figure 2).

### 3.3. Multivariable Analysis of the Association between BaPWV and the SRT Result

The crude analysis of the male participants showed that the SRT result and baPWV had an inverse association (≤3.10 cm [reference]; 3.11–11.00 cm: β = −84.05, 95% CI = −142.36–−25.76, *p* < 0.001; ≥11.01 cm: β = −121.72, 95% CI = −180.75–−62.68, *p* = 0.005). After the adjustment, the inverse association between a higher SRT result tertile and baPWV remained significant (3.11–11.00 cm [reference]: β = −74.45, 95% CI = −140.93–−8.55, *p* < 0.027; ≥11.01 cm, β = −108.17, 95% CI = −177.65–−38.70, *p* = 0.002).

Meanwhile, the crude analysis of the female participants did not show a significant relationship (≤14.00 cm [reference]; 14.01–20.60 cm: β = −13.41, 95% CI = −104.31–77.50, *p* = 0.773; ≥20.60 cm: β = −76.68, 95% CI = −167.59–14.23, *p* = 0.098). After the adjustment, there was no significant association found between the SRT result and baPWV (≤14.00 cm [reference]; 14.01–20.60 cm: β = 0.58, 95% CI = −70.69–71.85, *p* = 0.987; ≥20.60 cm: β = −5.26, 95% CI = −77.59–67.08, *p* = 0.887) (Table 3).

## 4. Discussion

In this study, we found that in the older Korean adults, the male sex showed a significant negative correlation with flexibility, expressed as the SRT result, and systemic arterial stiffness, expressed as the baPWV. In addition, the mean baPWV decreased with higher SRT performance, and this inverse association was significant. These findings remained significant even after adjusting for extensive confounding factors. Meanwhile, the female sex did not show any significant association between the SRT result and baPWV. Although there was a decreasing trend of the mean baPWV with higher SRT performance in older female adults, it was not significant. Furthermore, no linear association was found in the scatterplot and generalized linear analysis among the female participants.

The SRT is a validated measure of flexibility in young and older adults [19,20,21,22]. Previous studies have assessed the association between flexibility and systemic arterial stiffness using the SRT [2,9]. Yamamoto et al. showed that poor trunk flexibility, assessed as the SRT result, was significantly associated with greater systemic arterial stiffness, assessed as the baPWV [2]. They hypothesized that such a relationship results from the similarities of the compositions of the muscles and vascular structure, including elastin and collagen [2]. Although this study suggests a potential relationship between flexibility and arterial stiffness, this study result is limited, as only a small number of older adults were included (*n* = 132). In addition, although the study used different flexibility criteria, depending on the age and sex of the participants, it did not report the results based on sex. This could be another limitation of the study, as arterial stiffness might have different associations in each sex [23].

In this regard, Nishiwaki et al. addressed the difference in the association between flexibility and arterial stiffness in both sexes [9]. Among 1150 adults, the male sex showed an inverse association between flexibility and arterial stiffness, assessed using the cardio–ankle vascular index [9]. Conversely, the female sex showed an inverse association only in older women (60–89 years of age) [9]. Nishiwaki et al. interpreted this result as an effect of estrogen [9]. They then suggested that the vasodilatory and antiarteriosclerosis effects of estrogen attenuate the relationship in premenopausal women; thus, the correlation appears only in postmenopausal women [9,23].

Our study findings agree with the abovementioned study findings on the inverse association between flexibility and arterial stiffness among male participants. However, our study did not find any significant association between flexibility and arterial stiffness among older female adults. There are multiple possible explanations for this finding. First, testosterone and estrone levels increase from the age of 70 years in women [24]. It is known that estrogen production decreases arterial stiffness [25]. Estrogen receptors α and β are detected in human vasculature [25]. Estrogen is thought to preserve arterial compliance through these receptors by inducing vasodilation and vascular matrix formation [25,26]. In addition, recent studies have highlighted the protective role of testosterone in vascular stiffness [23]. Previous studies have reported that PWV and the circulating free testosterone index are inversely related. In addition, an arterial stiffness index calculated from the common carotid artery and simultaneous brachial artery blood pressure had a negative correlation with serum testosterone [27]. The levels of testosterone decline with age in males, with 35% of men in the seventh decade having lower testosterone levels than younger adults [28]. In our study cohort, the mean age of the male participants was in their 70s (73.05 ± 4.82, 71.29 ± 4.31, and 70.13 ± 3.68 years for the first, second, and third tertiles, respectively). Participants in the female lower STR result tertile were over 70 years (71.33 ± 4.14, 71.07 ± 4.00, and 69.50 ± 4.04 years for the first, second, and third tertiles, respectively). Compared with male participants, relatively increased estrogen and testosterone levels in female lower SRT result tertiles could have mitigated the relationship between flexibility and arterial stiffness.

Second, the female participants in our study were generally physically active. Even in the least flexible participants, 63.46% were physically active. According to a previous study on physical activity levels in a Korean population, 15.8% of individuals engaged in moderate and vigorous physical activities, and only 53.3% of female individuals over the age of 60 years achieved the age-recommended physical activity level [29]. Physically active individuals tend to have better flexibility [30]. The high physical activity level of our female participants might have mitigated the association between flexibility and the baPWV. Third, the results of the abovementioned previous study might have been affected by the relatively smaller number of included participants (*n* = 196). Fourth, the sex difference in the SRT is well known. However, flexibility measured by the SRT could be affected by spine curvatures and pelvic positions [31]. Thoracic kyphosis increases with age, particularly in older females [32]. A previous study has shown that when performing the SRT, the thoracic spine contributes more than the lumbar spine or hip joint [33]. This differential change of spinal curvature in older female adults could have affected the association between flexibility and baPWV. Lastly, the previous study did not incorporate blood data, including blood glucose and lipid levels [9]. As serum glucose and cholesterol levels affect arterial stiffness, the relationship between flexibility and arterial stiffness might have been affected in the previous study, resulting in different results from our study [9,34].

Our study is unique as it is adjusted for extensive confounding factors that can affect the relationship between flexibility and arterial stiffness, including age, blood pressure, heart rate, serum glucose level, LDL cholesterol level, history of smoking, and BMI, which are known to affect the baPWV [34]. In addition, we adjusted for the medical history of myocardial infarction and stroke, which can confound the association between flexibility and arterial stiffness [35,36]. Furthermore, the physical activity level is known to be associated with both flexibility and arterial stiffness [30,37]. We obtained more robust findings by incorporating the physical activity level in our analysis. Lastly, to our knowledge, we incorporated the largest number of older adults to assess the relationship between flexibility and arterial stiffness.

### Limitations

Despite these strengths, our findings should be interpreted with caution owing to the following limitations. First, our study is a cross-sectional design. Our results suggest a potential relationship between the SRT result and baPWV in older adults but cannot confirm the relationship. Second, our study incorporated a single race (Korean). As the SRT result can be influenced by body habitus, our findings should be cautiously applied to other ethnicities. Third, the waist circumference, which could affect the SRT result, was not incorporated in our analysis [38]. To mitigate this limitation, we adjusted for the BMI as a confounding factor, which is known to be significantly associated with waist circumference [39]. Lastly, information regarding underlying musculoskeletal conditions that can affect the SRT test result was unavailable. Those who could not perform the SRT due to medical conditions were not included in the study, so we believe the effect of this lack of information is minimal. However, it is possible that the performance of some of the participants was limited due to underlying musculoskeletal conditions. In addition, other underlying comorbidities that were not included in the analysis might have affected the study result. Future prospective studies that include diverse ethnicities are required to confirm our study findings.

## 5. Conclusions

Our study results suggest an inverse association between trunk flexibility and systemic arterial stiffness, expressed as the SRT result and baPWV, respectively, in older Korean men but no significant relationship in older Korean women. Thus, there may be a differential impact of sex on the relationship between flexibility and arterial stiffness in older adults.

## Figures and Tables

**Table 1:** Baseline characteristics.

	Male Participants					Female Participants				
		≤3.10 cm	3.11–11.00 cm	≥11.01 cm	*p*		≤15.00 cm	15.01–22.00 cm	≥22.01 cm	*p*
*n*	308	102	103	103		308	104	102	102	
Age (year)	71.44 ± 4.42	73.05 ± 4.82	71.29 ± 4.31	70.13 ± 3.68	<0.001	70.64 ± 4.13	71.33 ± 4.14	71.07 ± 4.00	69.50 ± 4.04	0.003
Height (cm)	166.12 ± 5.57	165.85 ± 5.29	166.30 ± 5.82	166.18 ± 5.58	0.820	152.64 ± 4.99	152.41 ± 4.63	152.24 ± 5.10	153.31 ± 5.19	0.231
Weight (kg)	66.04 ± 8.59	65.96 ± 9.07	65.13 ± 8.97	67.13 ± 7.59	0.198	57.70 ± 7.81	57.87 ± 8.59	57.81 ± 7.45	57.40 ± 7.37	0.887
Sit-and-reach test result (cm)	6.58 ± 9.97	−5.08 ± 6.83	6.86 ± 2.43	16.80 ± 4.62	<0.001	18.04 ± 7.48	9.63 ± 4.69	18.74 ± 2.29	25.92 ± 2.56	<0.001
Resting HR	62.69 ± 10.63	64.30 ± 10.60	61.71 ± 0.90	62.29 ± 11.71	0.006	64.15 ± 8.77	65.12 ± 9.24	63.87 ± 9.07	63.44 ± 7.92	0.364
BMI	23.87 ± 2.48	23.85 ± 2.63	23.60 ± 2.64	24.19 ± 2.11	0.218	24.70 ± 3.07	24.81 ± 3.27	24.90 ± 2.91	24.42 ± 3.03	0.492
LDL cholesterol level	116.95 ± 33.94	113.83 ± 31.85	118.75 ± 32.00	117.82 ± 37.73	0.559	131.35 ± 35.53	131.47 ± 36.82	125.29 ± 34.63	137.29 ± 34.38	0.054
Glucose level	102.73 ± 25.01	104.70 ± 36.44	104.29 ± 19.79	99.25 ± 15.50	0.224	101.91 ± 21.50	105.63 ± 25.03	100.55 ± 17.05	99.46 ± 21.29	0.088
sBP	130.72 ± 15.04	129.90 ± 14.74	129.12 ± 16.28	133.21 ± 16.66	0.112	133.72 ± 16.54	133.44 ± 14.57	134.56 ± 17.20	133.17 ± 17.82	0.817
dBP	74.04 ± 8.92	72.87 ± 9.73	73.56 ± 8.40	75.61 ± 8.55	0.077	74.09 ± 9.20	72.47 ± 9.05	75.01 ± 9.73	74.57 ± 8.84	0.11
History of smoking	22 (7.10)	4 (4.30)	11 (9.82)	7 (6.25)	0.307	4 (1.30)	2 (1.9)	2 (1.9)	0 (0)	0.367
Active lifestyle	130 (42.20)	34 (36.55)	40 (35.71)	56 (50)	0.009	190 (61.69)	66 (63.46)	64 (62.74)	60 (58.82)	0.763
History of HTN	120 (38.96)	33 (35.48)	42 (37.50)	45 (40.18)	0.463	138 (44.80)	48 (46.15)	54 (52.94)	36 (35.29)	0.038
History of dyslipidemia	52 (16.88)	13 (13.98)	16 (14.29)	23 (20.54)	0.194	102 (33.12)	34 (32.69)	42 (41.18)	26 (25.49)	0.058
History of stroke	20 (6.49)	5 (5.38)	8 (71.43)	7 (6.25)	0.867	11 (3.57)	5 (4.80)	3 (2.94)	3 (2.94)	0.706
History of MI	10 (3.25)	4 (4.30)	4 (3.57)	2 (1.79)	0.63	7 (2.27)	2 (1.90)	1 (1.00)	4 (3.92)	0.355

Participants’ baseline characteristics are expressed according to SRT tertile. Continuous variables are expressed as means ± standard deviations. Categorical variables are expressed as frequencies (%). HR, heart rate; BMI, body mass index; LDL, low-density lipoprotein; sBP, systolic blood pressure; dBP, diastolic blood pressure; HTN, hypertension; MI, myocardial infarction.

**Table 2:** The trend of the baPWV according to the sit-and-reach test result tertiles.

	Male Participants					
	Total	≤3.10 cm	3.11–11.00 cm	≥11.01 cm	*p*	*p* for trend
*n*	308	102	103	103		
baPWV, cm/s	1635.64 ± 283.52	1713.49 ± 331.31	1608.04 ± 260.95	1595.36 ± 246.63	0.006	0.003
	**Female Participants**					
	Total	≤15.00 cm	15.01–22.00 cm	≥22.01 cm	*p*	*p* for trend
*n*	308	104	102	102		
baPWV, cm/s	1635.09 ± 333.89	1663.54 ± 344.91	1652.23 ± 358.52	1588.95 ± 292.79	0.227	0.109

Data are expressed as means ± standard deviations. baPWV, brachial–ankle pulse wave velocity.

**Table 3:** Association between the sit-and-reach test result and brachial–ankle pulse wave velocity.

		Male Participants				Female Participants		
		β	95% CI	*p*		β	95% CI	*p*
Crude	≤3.10 cm (Ref)	Ref	Ref	Ref	≤14.00 cm (Ref)	Ref	Ref	Ref
	3.11–11.00 cm	−84.05	−142.36–−25.76	<0.001	14.01–20.60 cm	−13.41	−104.31–77.50	0.773
	≥11.01 cm	−121.72	−180.75–−62.68	0.005	≥20.60 cm	−76.68	−167.59–14.23	0.098
Adjusted model	≤3.10 cm (Ref)	Ref	Ref	Ref	≤14.00 cm (Ref)	Ref	Ref	Ref
	3.11–11.00 cm	−74.45	−140.93–−8.55	0.027	14.01–20.60 cm	0.58	−70.69–71.85	0.987
	≥11.01 cm	−108.17	−177.65–−38.70	0.002	≥20.60 cm	−5.26	−77.59–67.08	0.887

Adjusted model: Adjusted for age, history of smoking, physical activity level, history of hypertension, history of dyslipidemia, history of stroke, history of myocardial infarction, body mass index, serum glucose level, serum low-density lipoprotein cholesterol level, systolic blood pressure, and heart rate at rest. Ref, reference; CI, confidence interval.

## Data Availability

Unavailable as an initial agreement form for study participation stated that the participants’ data will not be shared.
